# Comparative Genomics of *Xylella fastidiosa* Explores Candidate Host-Specificity Determinants and Expands the Known Repertoire of Mobile Genetic Elements and Immunity Systems

**DOI:** 10.3390/microorganisms10050914

**Published:** 2022-04-27

**Authors:** Guillermo Uceda-Campos, Oseias R. Feitosa-Junior, Caio R. N. Santiago, Paulo M. Pierry, Paulo A. Zaini, Wesley O. de Santana, Joaquim Martins-Junior, Deibs Barbosa, Luciano A. Digiampietri, João C. Setubal, Aline M. da Silva

**Affiliations:** 1 Departamento de Bioquímica, Instituto de Química, Universidade de São Paulo, São Paulo 05508-000, Brazil; gucedac@ime.usp.br (G.U.-C.); oseias.rf.junior@gmail.com (O.R.F.-J.); pmpierry@gmail.com (P.M.P.); wesleyoliveira@pcs.uespi.br (W.O.d.S.); martins.jr.bio@gmail.com (J.M.-J.); ertablo@gmail.com (D.B.); setubal@iq.usp.br (J.C.S.); 2 Programa de Pós-Graduação Interunidades em Bioinformática, Universidade de São Paulo, São Paulo 05508-090, Brazil; caio.rns@gmail.com; 3 Department of Plant Sciences, University of California, Davis, CA 95616, USA; pazaini@ucdavis.edu; 4 Escola de Artes, Ciências e Humanidades, Universidade de São Paulo, São Paulo 03828-000, Brazil; digiampietri@usp.br

**Keywords:** phytopathogen, virulence, pangenome, prophage, phage-defense, Xanthomonadaceae

## Abstract

*Xylella fastidiosa* causes diseases in many plant species. Originally confined to the Americas, infecting mainly grapevine, citrus, and coffee, *X. fastidiosa* has spread to several plant species in Europe causing devastating diseases. Many pathogenicity and virulence factors have been identified, which enable the various *X. fastidiosa* strains to successfully colonize the xylem tissue and cause disease in specific plant hosts, but the mechanisms by which this happens have not been fully elucidated. Here we present thorough comparative analyses of 94 whole-genome sequences of *X. fastidiosa* strains from diverse plant hosts and geographic regions. Core-genome phylogeny revealed clades with members sharing mostly a geographic region rather than a host plant of origin. Phylogenetic trees for 1605 orthologous CDSs were explored for potential candidates related to host specificity using a score of mapping metrics. However, no candidate host-specificity determinants were strongly supported using this approach. We also show that *X. fastidiosa* accessory genome is represented by an abundant and heterogeneous mobilome, including a diversity of prophage regions. Our findings provide a better understanding of the diversity of phylogenetically close genomes and expand the knowledge of *X. fastidiosa* mobile genetic elements and immunity systems.

## 1. Introduction

*Xylella fastidiosa* is a Gram-negative bacterium in the Xanthomonadaceae family that colonizes the xylem vessels of its plant hosts and is exclusively vectored by xylem sap-feeding hemipteran insects [1,2]. This bacterium causes several crop diseases, such as Pierce’s disease (PD) of grapevine [3], citrus variegated chlorosis (CVC) [4], coffee leaf scorch (CLS) [5], plum leaf scald (PLS) [6], and olive quick decline syndrome (OQDS) [7]. While *X. fastidiosa* has also been associated with diseases in many other plant species, the bacterium behaves as a commensal endophyte in a variety of its plant hosts [8,9].

A range of pathogenicity and virulence factors has been identified in *X. fastidiosa* that potentially enable the bacterium to overcome host defenses and successfully establish itself in the xylem tissue [1,9,10]. *X. fastidiosa* cells form biofilm-like structures that are crucial for successful acquisition and transmission by the insect vectors as well as for plant host colonization and pathogenesis [1,11]. Progression of the disease symptoms is associated with *X. fastidiosa* systemic spread through the xylem vessel network which requires dispersal of bacterial cells from the biofilms [12,13,14,15] as well as twitching motility [16] and degradation of pit membranes by bacterial cell wall–degrading enzymes (CWDEs) [17,18]. Moreover, the severity of symptoms is exacerbated by host-derived xylem occlusions (i.e., tyloses) elicited by *X. fastidiosa* colonization of grapevine [19]. Indeed, the symptoms caused by *X. fastidiosa* infection are suggestive of hydric stress and vary in intensity depending on pathogen genotype, plant host species/genotype, plant age, cultivation practices, and environmental conditions [10,20].

Originally confined to the Americas, *X. fastidiosa* has spread to various plant species in a number of European countries, possibly through the importation of infected plant material [9,21,22]. Currently, most of *X. fastidiosa* strains are categorized in three major subspecies, *fastidiosa*, *pauca* and *multiplex,* which are presumed to have originated in Central America (subsp. *fastidiosa*), South America (subsp. *pauca*) and North America (subsp. *multiplex*) [8,9,23]. Another two subspecies (subspp. *sandyi* and *morus*) native to North America have also been proposed [24,25]. Furthermore, *X. fastidiosa* strains can be classified into sequence types (STs) based on a multilocus sequence typing (MLST) scheme with seven housekeeping genes [26,27].

There is a loose association of *X. fastidiosa* subspecies or STs with host specificity, yet some strains can infect multiple hosts [10,28]. Indeed, intersubspecific homologous recombination has been associated with *X. fastidiosa* adaptation to novel hosts [25,29,30]. However, the mechanisms by which the distinct *X. fastidiosa* strains successfully colonize specific plant hosts remain unclear. *X. fastidiosa* lacks the Type III secretion system (T3SS) [31], a membrane-embedded nanomachine typical of Gram-negative pathogens, which delivers effector proteins directly into host cells triggering or suppressing defense mechanisms, respectively in resistant or susceptible plants [32]. Instead, *X. fastidiosa* type II secretion system (T2SS) seems to be a relevant delivery apparatus of its virulence proteins [10,15,33,34]. It has been suggested that compatibility between xylem pit membrane carbohydrate composition and *X. fastidiosa* T2SS-secreted cell wall degrading enzymes is necessary for disease progression [35]. Moreover, since *X. fastidiosa* lipopolysaccharide (LPS) long chain O-antigen effectively delays plant innate immune recognition in grapevine, the heterogeneity of O-antigen composition may be among the mechanisms underlying *X. fastidiosa* host range [36].

Comparative genomics studies of *X. fastidiosa* strains isolated from different plant hosts and from diverse geographical regions identified shared and exclusive genes among these strains, chromosome rearrangements, indels, single nucleotide polymorphisms (SNPs) as well as differences in their mobile genetic elements (MGE) repertoire, such as plasmids, genomic islands and prophages [21,29,30,37,38,39,40,41,42,43,44,45,46,47,48]. While some studies suggest that strains belonging to a phylogenetic group have similar pathogenicity mechanisms and strong selection, possibly driven by host adaptation [42,43], other studies identified differences in each subspecies, such as enriched molecular functions [45] and distinct rates and events of recombination [21,29,30,48].

The availability of new whole genome sequences of *X. fastidiosa* strains from diverse plant hosts and distinct geographical regions fosters up-to-date comparisons to be made. Here we present a thorough comparative analysis of 94 *X. fastidiosa* genomes with the goal of providing insights into host specificity determinants for this phytopathogen as well as expanding the knowledge of its MGE content and of its immunity systems.

## 2. Materials and Methods

### 2.1. Data Collection, Curation, and Annotation

A collection of 132 *X. fastidiosa* genome assemblies were downloaded from National Center for Biotechnology Information (NCBI) GenBank database [49] (https://www.ncbi.nlm.nih.gov/genome/genomes/173) accessed in 19 July 2021 (Table S1). This collection was curated with an in-house workflow which is now available at Github repository (https://github.com/gucedac/uceda-campos-etal-2022-microorganisms) and depicted in Figure S1 to remove genomes of laboratory variants, redundancies, and assemblies with contamination ≥5%, or with ≥1% of ambiguous bases, or with less than 20 tRNA genes or missing any of the 3 rRNA genes. Contamination and completeness of genome assemblies were evaluated using CheckM software [50]. Ambiguous bases in the assemblies were evaluated using QUAST tool [51]. Genomes that were not selected in the first curation round but represented a non-redundant strain, host or geographical region and had an associated publication were retrieved and included in the final curated collection, making a total of 94 genome assemblies (Table S1). This curated genome collection was annotated using Prokaryotic Genome Annotation Pipeline (PGAP) [52] standalone software package (https://github.com/ncbi/pgap), release 2021-07-01.build5508, accessed in 2 August 2021.

### 2.2. Genome Comparisons

Comparative genomics analyses, pangenome, core genome and accessory genome reconstruction were performed using the Gene Tags Assessment by Comparative Genomics (GTACG) framework (https://github.com/caiorns/GTACG-backend) accessed in 10 September 2021. GTACG is based on an algorithm that uses clustering coefficient to find and maximize the number of orthologous groups in genomes from closely related strains [53]. The PGAP annotated genomes were uploaded in GTACG framework, and the protein coding sequences (CDSs) were compared using standalone BLASTP tool [54] with an e-value threshold of 1 × 10^−10^. The clustering tool in GTACG framework was used to find a threshold that maximizes the cluster coefficient of each cluster. We found that a threshold of 45% of the alignment length was enough to produce concise homologous clusters. Scripts and parameters used in these comparative analyses are available at https://github.com/gucedac/uceda-campos-etal-2022-microorganisms. Metadata information of the *X. fastidiosa* strains (Table S1) such as plant host and country of isolation was retrieved from NCBI BioSample database (*host* and *geo_loc_name* attributes) accessed in 19 July 2021. Sequence type (ST) of some *X. fastidiosa* strains was retrieved from public databases for molecular typing and microbial genome diversity (PubMLST) [27] and from the literature. For strains not deposited in the PubMLST, ST was identified by running BLASTN to identify the MLST loci [26] in the respective *X. fastidiosa* genome followed by analyses of the alleles in the PubMLST database.

### 2.3. Phylogenetic Analyses

Nucleotide sequences of core genome orthologous CDSs were aligned using Clustal Omega v.1.2.1 [55] with default parameters. Then, the sequences were concatenated and homologous recombination regions were masked using Gubbins v.3.1.6 [56]. The core genome phylogenetic tree was built with a maximum-likelihood (ML) method using IQ-TREE v.1.5.4 [57] with a model predicted by ModelFinder and an ultrafast bootstrap of 1000 replicates [58].

Phylogenetic trees for 1605 orthologous CDSs found in more than 80 strains including the soft-core and core genomes were built with a maximum-likelihood (ML) method using IQ-TREE v.1.5.4 [57] with an ultrafast bootstrap of 1000 replicates. Information of plant host of origin for the strains was mapped to the conserved CDSs phylogenetic trees and a *Score of mapping* (*Smap*) was estimated. The overall concept behind *Smap* was based on consenTRAIT, a metric that estimates the clade depth where organisms share a trait [59]. The *Smap* for each phylogeny was estimated using a custom Python script that uses Phylo module to find clades in a tree and to calculate the proportion of each plant host in each clade (https://github.com/gucedac/uceda-campos-etal-2022-microorganisms). The highest proportions of a given host is then retrieved and summed to obtain the *Smap*. We calculated *Smap* for both ML and bootstrap trees to get the average of *Smap* and the percentage of the trees with the same *Smap* to retrieve the confidence level. *Smap* values close to 1 indicate a strong relationship between specific hosts and the phylogenetic tree of an orthologous CDS while lowest values (~0.1) are found for highly conserved CDSs unrelated to specific hosts.

### 2.4. Functional Annotation

Orthologous protein clusters encoded by the core, accessory and singleton genomes were compared to the Clusters of Orthologous Groups (COGs) [60] database using rpsblast+ (BLAST version 2.9.0) [61], with a cut-off e-value of 1 × 10^−6^. COG categories were assigned to the best hits of rpsblast+ analysis.

### 2.5. Mobile Genetic Elements Prediction

Mobile Genetic Elements (MGE), such as prophages, genomic islands (GI) and insertion sequences (IS) were identified in the genome assemblies by a combination of prediction tools coupled with manual curation as previously described [62]. Prophage regions were predicted with Virsorter2 [63] and PHASTER [64]. Inovirus_detector software (https://github.com/simroux/Inovirus) accessed in 4 November 2021 was used for identification of prophages from the Inoviridae family (filamentous single-stranded DNA phages) [65]. GI regions were defined using SeqWord Sniffer [66] and GIPSy [67] software, which was used to assign one or more categories related to GI potential function. GI regions overlapping to prophage regions were not considered. IS regions were predicted using the ISEScan [68] software.

Retrieved prophage, GI, and IS nucleotide sequences were compared to explore homology relationships using BLAST all-vs-all. Results of BLAST with an identity and coverage alignment higher than 50% and 80%, respectively, were filtered, analyzed and the resulting sequence similarity network (SSN) was visualized with Cytoscape 3.8 software [69]. Taxonomic classification of intact and incomplete prophages according to PHASTER [64] output was performed with vContact2 [70] and with PhaGCN [71].

### 2.6. Prospection of Anti-MGE Defense Systems

Prokaryotic Antiviral Defence LOCator (PADLOC) tool [72] and Hidden Markov Models (HMM) profiles built in-house were used to analyze known antiphage defense systems such as Restriction-Modification (R-M), Disarm, Brex, pAgos, DND, Abortive Infection (Abi), Hachiman, Shedu, Septu, Lamassu, Druantia, CBASS, Gabija, Zorya and Wadjet [73,74,75]. To create HMM profiles, we recovered FASTA files with the amino acid sequences of each system retrieved from NCBI and IMG/M (Integrated Microbial Genomes & Microbiomes) databases [76] using the information available in the publication of the respective immune system against phages. PICI elements (Phage-inducible chromosomal islands) were searched in *X. fastidiosa* genomes using an in-house pipeline that enables detection of the main PICI features [77]. CRISPR-Cas systems were searched with the software CRISPRCasTyper (https://github.com/Russel88/CRISPRCasTyper) accessed in 22 November 2021) [78]. Scripts used for prospection of defense systems are available at https://github.com/gucedac/uceda-campos-etal-2022-microorganisms.

## 3. Results

### 3.1. General Features of X. fastidiosa Genomes

A set of 94 genome assemblies (Table S1) was selected from 132 *X. fastidiosa* genomes available in NCBI GenBank genomes database, following a workflow (Figure S1) that removed redundancies, genomes of laboratory variants and poor assemblies. The selected genomes are high-quality draft sequences [79] since they present high completeness (>98%) and low contamination (<1.45%) according to CheckM [50] analysis. The average chromosome size of the selected 94 assemblies is 2,537,252 bp ± 90,235 bp with an average GC content of 51.88% ± 0.36. Strains Hib4 (isolated from *Hibiscus* spp.) and Griffin-1 (isolated from *Quercus rubra*) have, respectively, the largest (2,813,286 bp) and smallest (2,387,314 bp) chromosome sizes. While 46 strains of the selected genome assemblies do not include plasmid related-contigs, the plasmid numbers in the other strains are 1 (34 strains), 2 (9 strains), and 4 (5 strains), which include conjugative and mobilizable as well as non-mobilizable plasmids [46]. Chromosomes of the selected genomes have 2291 ± 131 CDS and 110 ± 45 protein-coding pseudogenes annotated by PGAP [52]. These results indicate a reasonable homogeneity in the genomes of distinct *X. fastidiosa* strains concerning their chromosome sizes and GC content. In contrast, the plasmid content shows a greater diversity among strains consistent with previous observations [46].

### 3.2. X. fastidiosa Pan and Core Genomes

**Figure 1:**
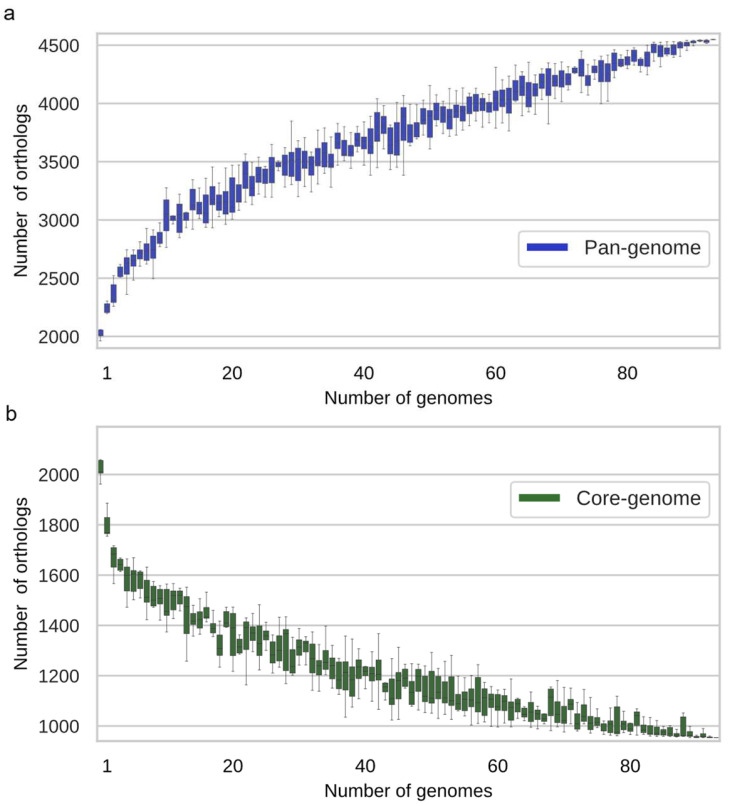
Pangenome and core-genome of 94 *X. fastidiosa* strains. Pangenome (**a**) and core genome (**b**) curves. Each boxplot represents the distribution of the number of orthologous CDSs clusters added (pangenome) or in common (core genome) with the addition of new genomes.

The pangenome of *X. fastidiosa* (number of orthologous CDSs clusters present in the 94 genomes) was calculated using the GTACG framework [53], considering chromosome and plasmids CDSs, since pangenomes are composites of the host chromosome together with the MGEs [80]. The *X. fastidiosa* pangenome growth curve has not yet reached saturation (Figure 1a) and comprises 4549 orthologous CDSs while the core genome (conserved orthologous CDSs present in all 94 genomes) is composed of 954 CDSs (Figure 1b). The trend of the curve (Figure 1b) suggests that a slight decrease in the core genome might happen as more *X. fastidiosa* genome assemblies are compared. Calculation of the soft-core genome (conserved orthologous CDSs present 95% of the selected genomes, i.e., 89 genomes) showed 1567 CDSs (34.4% of the pangenome).

We found that the vast majority (90%; 64/71) of the CDSs previously identified or predicted to be virulence and pathogenicity factors for *X.* *fastidiosa* [10,33,36,38,81,82,83,84] belong either to the core or soft-core genomes (Table S2). The lack of some of these CDSs in some strains is mostly due to pseudogenization, as in the case of polygalacturonase ortholog (PD1485 in Temecula1 strain), which carries a frameshift mutation [38] in strains from subspecies *pauca* isolated from citrus (strains 9a5c, U24D, Fb7, J1a12, B111, CVC0251, CVC0256, 11399 and XRB), coffee (strains 32 and 3124), and vinca (strain CFBP8078). Other strains from subspecies *pauca* such as Pr8x, 6c, Hib4, COF0324, CFBP8072, CODIRO, and De Donno harbor an intact polygalacturonase sequence, similarly to all other strains analyzed in this study from subsp. *multiplex* and *fastidiosa*. Polygalacturonase has been shown to be a critical virulence factor for *X. fastidiosa* pathogenesis in grapevine [17]; therefore, it is possible that another cell wall-degrading enzyme, such as the putative pectin-lyase (PD0090 in Temecula1 strain) [85], may perform that function in the strains that carry the frameshift mutation.

### 3.3. Genome-Scale Phylogeny

**Figure 2:**
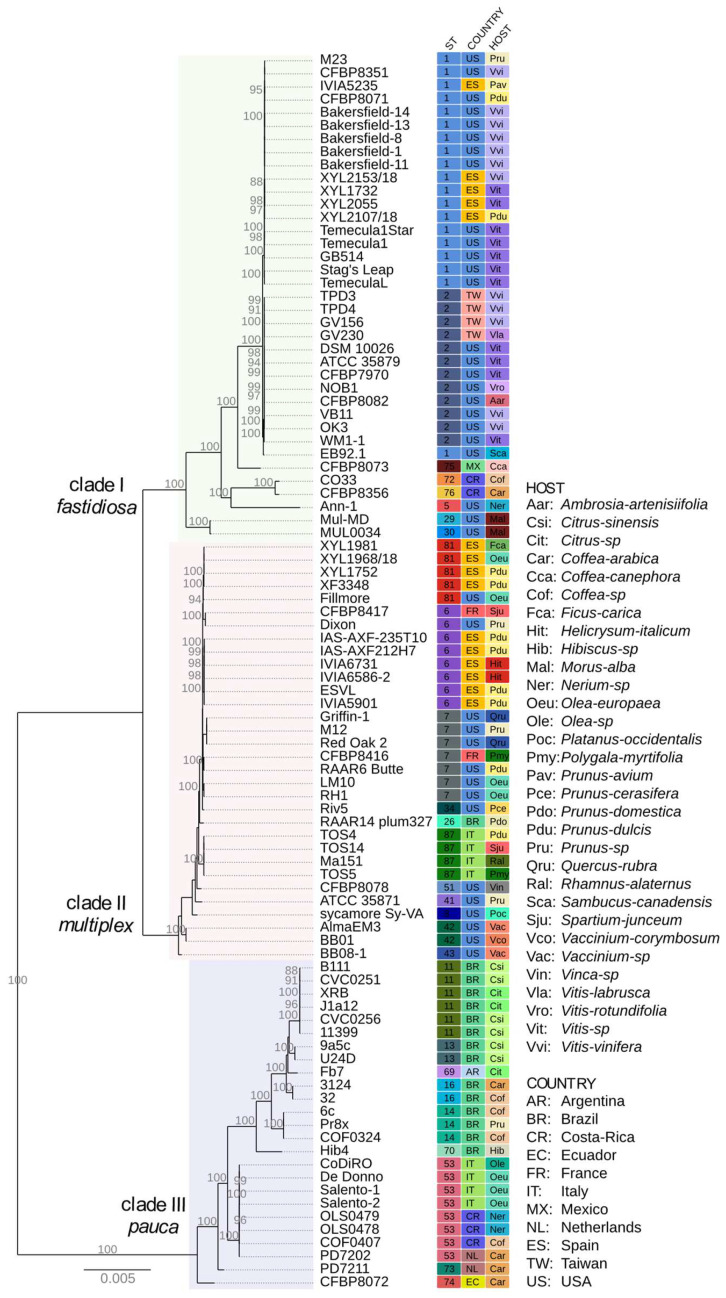
Core genome-scale phylogeny. Nucleotide sequences of *X. fastidiosa* core genome CDSs with homologous recombination regions masked from 94 strains were used for a Maximum Likelihood (ML) phylogenetic reconstruction. The three major clades grouped strains from subspecies *fastidiosa, multiplex* and *pauca*. Information of sequence type (ST), country of isolation and plant host of origin for each strain as listed in Table S1 are shown within the colored squares according to the indicated abbreviations.

The nucleotide sequences of the core genome (954 orthologous CDSs) were used for a genome-scale phylogeny. Since homologous recombination can impact *X. fastidiosa* phylogenies [86], these regions were masked in these CDSs, even though this resulted in loss of resolution within-subspecies branches (Figure 2). The Maximum Likelihood (ML) tree with homologous recombination regions masked (Figure 2) grouped the 94 *X. fastidiosa* strains in three major clades defined by strains from the subspecies *fastidiosa* (clade I)*, multiplex* (clade II)*,* and *pauca* (clade III). The strains from subspecies *morus* (Mul-MD and MUL0034) and *sandyi* (Ann-1, CO33 and CFBP8356) grouped in subclades of the major clade I. The overall topology of this core-genome-based phylogeny tree agrees with previously reported genome-wide phylogenies *X. fastidiosa* strains [43,86] and with a *k-mers* based phylogeny of 72 *X. fastidiosa* strains [30].

Information of ST, country of isolation and host of origin for each strain (Table S1) integrated to the genome-scale phylogeny (Figure 2) show that several subclades have members sharing same country instead of host of isolation. For example, subclade formed by strains of ST1 (except EB92.1) belonging to subsp. *fastidiosa* has members isolated from USA and Spain. Another subclade of subsp. *fastidiosa*, formed by strains of ST2, has only members from USA and Taiwan. Subclades of strains with ST6, ST7 and ST87 belonging to subsp. *multiplex* have members mainly from Spain, USA, and Italy, respectively. Subclades of strains with ST11, ST14 and ST53 were distributed among subspecies *pauca*, of which the first two subclades have members isolated from Brazil while subclade ST53 has members isolated from Costa Rica and Italy. Strains from Mexico and Ecuador, respectively with ST75 and ST74, were found in a branch by themselves.

### 3.4. Exploring Candidates to Host-Specificity Determinants

The relationship of the orthologous clusters of 1605 CDSs found in more than 80 strains with their respective plant host of origin was explored by mapping the host metadata to the individual phylogenies. A Score of mapping (*Smap*) was estimated where *Smap* close to 1 indicates a strong relationship between the hosts and the phylogenetic tree of each orthologous CDS. Among 1605 phylogenies analyzed (Table S3), the lowest *Smap* values (~0.14) were for highly conserved CDSs such as ribosomal (PD2123) and cell division (PD1872) proteins. On the other hand, the highest *Smap* values found were ~0.5 belonging to CDSs encoding TonB-dependent receptor and the hypothetical protein PD0014 (Table S3). Only 9 orthologous CDSs previously identified or predicted to be virulence and pathogenicity factors were among the 100 CDSs with *Smap* values greater than 0.44 with confidence >90% (Table S3). These 9 CDSs include two related to adhesion (PD0058, PD0528), two related to polysaccharide hydrolysis (PD0529, PD1833), two related to polysaccharide synthesis (PD0815, PD1801) and three that encode, respectively, quorum sensing response regulator (PD0406), multidrug efflux pump (PD1403) and lipase/esterase (PD1211). However, we reasoned that these medium *Smap* scores do not provide strong support to consider these CDSs as candidates to host specificity determinants.

### 3.5. Unraveling X. fastidiosa Accessory Genome and Its Mobile Genetic Elements

**Figure 3:**
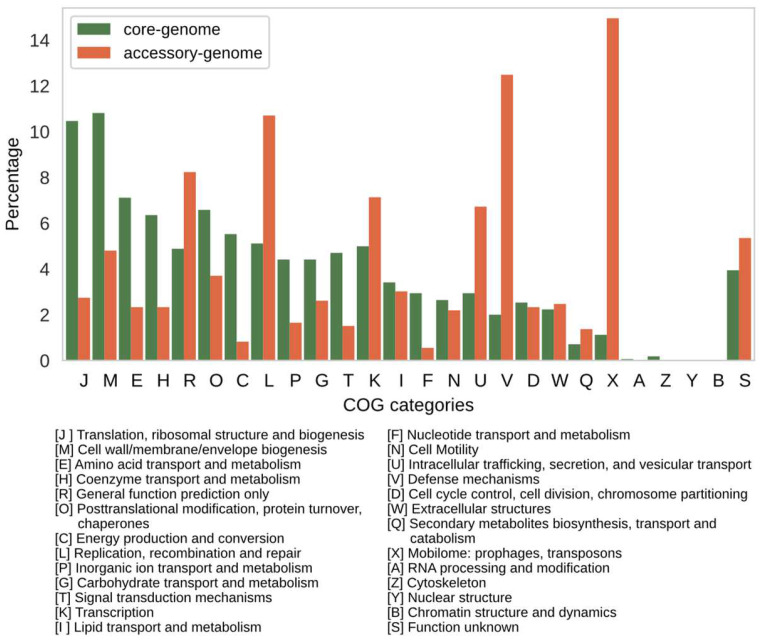
Profile of Clusters of Orthologous Groups (COG) functional categories in the core and accessory genomes of the 94 assemblies of *X. fastidiosa* strains. Distribution (percentage) of COG categories in the core (green bars) and accessory (orange bars) genomes.

The distribution of core and accessory genomes of the 94 strains among COG functional categories is depicted in Figure 3. As expected, the COG functional categories of highly conserved biological processes, such as “Translational, ribosomal structure, and biogenesis” (category J), and “Cell wall/membrane/envelope biogenesis” (category M) comprise a substantial fraction of the core genome in comparison to the accessory genome. In contrast, the accessory genome which comprises 2219 CDSs is enriched in category X (Mobilome: prophages, transposons) that makes up ~15%. Other categories also enriched in the accessory genome are “Replication, recombination and repair” (category L) and “Defense mechanisms” (category V) which is suggestive of the ability of *X. fastidiosa* strains to cope with stress conditions in distinct environments.

**Figure 4:**
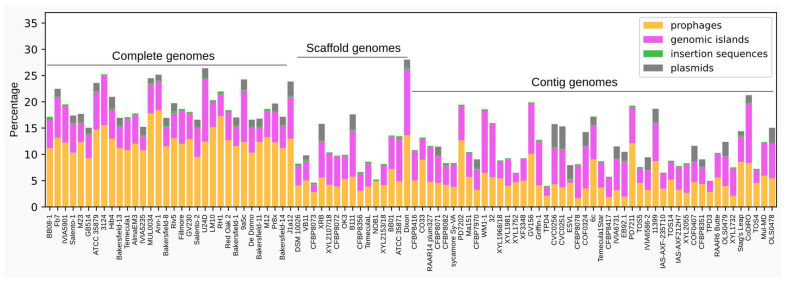
Percentage of mobile genetics elements distributed among the *X. fastidiosa* strains according to their genome assembly status: complete, scaffold and contig.

The enrichment of accessory genome with mobilome-associated CDSs prompted us to explore the full set of MGEs (prophages, genomic islands, insertion sequences and plasmids) in *X. fastidiosa* strains. Using a combination of prediction tools, we identified a comprehensive set of sequences related to the MGEs in the 94 genome assemblies analyzed here. The content of MGEs varies considerably among the strains, ranging from 3.8% to 27.76% of the genome, with a mean value of 13.92% ± 5.77%. Among the strains with the higher MGE content are Dixon, U24D, 3124, Ann-1, MUL0034 and 9a5c (Figure 4). It is important to note that the strains whose genome assemblies are in contigs showed the lower percentages of MGE content than the strains with complete genomes, possibly due to a reduced efficiency of the programs to predict MGEs in fragmented genomes. Overcoming this limitation will have to wait for the availability of complete versions of these genomes which, in most cases, requires resequencing with long-read technologies [87].

*X. fastidiosa* genome assemblies harbor 11.6 ± 2.71 prophage-related regions. Among the complete genomes, the strains RH1 and LM10 of subspecies *multiplex* have the greatest number of prophage regions (19 and 18, respectively) while those with the least prophage regions are the subspecies *pauca* strains Pr8x, Salento-2, De Donno (9, 9 and 10, respectively). We found 5 intact, 2 incomplete, 1 questionable and 3 remnant prophages in 9a5c strain (subsp. *pauca*), and 4 intact, 5 incomplete, 3 questionable and 1 remnant prophages in Temecula1 strain (subsp. *fastidiosa*). The genomes of *X. fastidiosa* also harbor on average 6.47 ± 2.57 genomic island regions. The strains U24D and 9a5c (subsp. *pauca*) have the greatest number of genomic islands (14 and 12, respectively) while the strains IVIA5235 and Bakersfield-11 (subsp. *fastidiosa*) have only 5 regions each. We found on average 6 ± 1.53 insertion sequences within certain prophages, genomic islands, chromosomes, or, occasionally, in plasmids.

**Figure 5:**
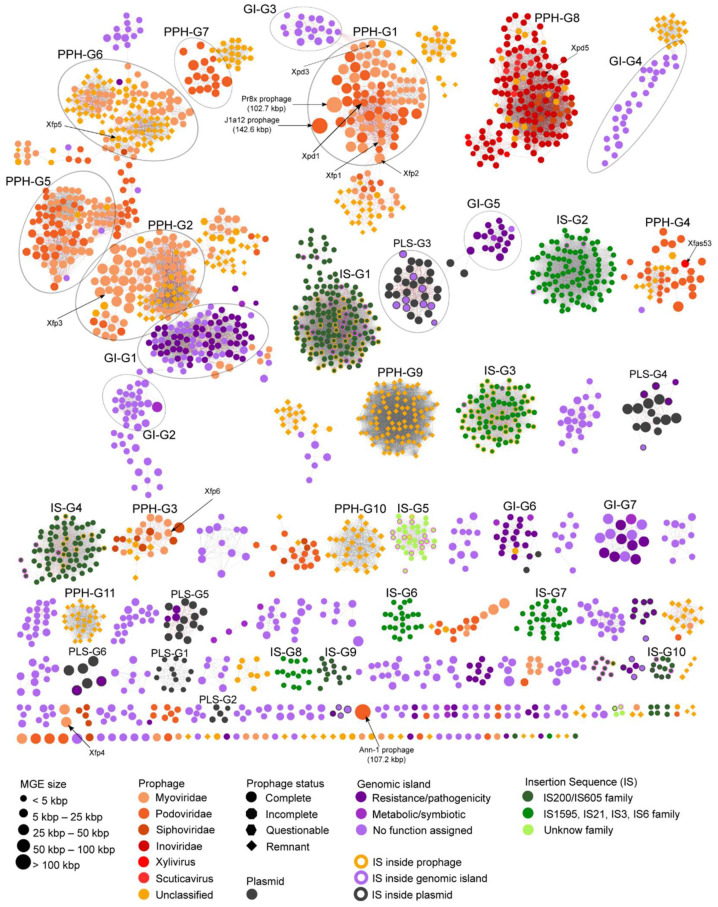
Sequence Similarity Network of the *X. fastidiosa* mobilome. The distinct MGEs (prophages (PPH), genomic islands (GI), insertion sequences (IS) and plasmids (PLS)) predicted in the 94 genome assemblies analyzed are indicated by the symbols (nodes) and the color code indicated on the bottom of the figure. Edges (lines connecting the symbols) represent the similarity of nucleotide sequence with an identity and coverage alignment higher than 50% and 80%, respectively. Symbol sizes shown in the bottom left represent MGE length in kbp. Prophage families and status are represented by different color circles and different shapes, respectively. Symbols of MGEs carrying ISs are outlined in orange (prophage), purple (genomic island) and dark gray (plasmid). PPH-G, GI-G, IS-G and PLS-G refer to the distinct groups highlighted in this work corresponding to prophages, genomic islands, insertion sequences, and plasmids, respectively. Previously reported prophage sequences are indicated (ex: Xfp6). Details of the MGEs pictured in the network are listed in Table S4.

The relationships between all predicted MGEs (Table S4) in *X. fastidiosa* genome assemblies were investigated by grouping them on a sequence similarity network (SSN) (Figure 5). The lengths of these MGE sequences vary from ~4.1 kbp to 142.6 kbp for prophages, ~3.5 kbp to 79.7 kbp for genomic islands, 112 bp to 2.5 kbp for insertion sequences, and ~1.2 kbp to 64.3 kbp for plasmids (Table S4). The SSN shows that prophage groups (PPH-G) present more connections than genomic island groups (GI-G), which are shown in small independent groups. On the other hand, most of insertion sequence groups (IS-G) have tight connections showing the conservation of this type of MGE in *X. fastidiosa* strains. Of all plasmid groups (PLS-G) shown in the SSN, only PLS-G1 and PLS-G2 have unique plasmid sequences while the others cluster with genomic island sequences. Only IS-G1 and IS-G2 belong to the core and soft-core genomes, respectively. The PPH-G and GI-G encompassing more strains were PPH-G9 (group of remnant prophages with integrases only) with 87 strains, and GI-G1 with 40 strains (Table S4). Several unique MGEs belonging to a single strain are shown at the bottom of Figure 5.

The intact prophages of the strains 9a5c (previously reported as Xfp1, Xfp2, Xfp3, Xfp4, and Xfp6), Temecula1 (Xpd1, Xpd3 and Xpd5), and 53 (Xfas53, isolated prophage) [88,89] are indicated in the SSN (Figure 5). The Xfp1, Xfp2, Xpd1 and Xpd3 were grouped into the PPH-G1, a group composed of intact prophages that curiously includes some classified as Myoviridae and others classified as Podoviridae (Figure S2a). Xfp3, Xfp5 and Xfp6 were grouped in the PPH-G2, PPH-G6 and PPH-G3, respectively. Prophages of PPH-G2 are mainly intact sequences classified as Myoviridae and PPH-G6 has prophages classified as Myoviridae and unclassified remnant prophages. Based on the taxonomic predictions, the Siphoviridae family was the least abundant family among the *X. fastidiosa* Caudovirales prophages (PPH-G3). The podovirus Xfas53 was found in the PPH-G4 having direct links with other six intact prophages of strains BB08-1, Riv5, MULL0034, RH1 and Ann-1, the latter harboring two Xfas53-like prophages that suggests a case of superinfection. A closer examination of the prophage groups, except PPH-G6, reveals that most of these sequences carry lysozyme and holin genes, commonly found in temperate and lytic bacteriophages. Other relevant CDSs found are those related to pathogenesis and virulence such as multidrug efflux RND transporter, found in some prophages of group PPH-G5 detected in Temecula1, M23, among other strains (Figure S2b); toxin-antitoxin proteins, such as RelE/ParE, HicB/HicA, MqsA family toxins; transposases and virulence factors found in sequences of groups PPH-G1, PPH-G2 and PPH-G3. Some prophage groups (PPH-G9, PPH-G10 and PPH-G11) contain remnant prophages that encode integrase fragments. It is worth noting that one of the larger prophages (107.2 kbp) belongs to Ann-1 and has no counterparts in the other strains analyzed here (Figure 5), but it is present in the just released genome of *X. fastidiosa* strain OC8 (GenBank assembly accession GCA_021474225.1).

Most of the prophages from group PPH-G8 were classified as Inoviridae [65]. This group encompasses 143 sequences from 80 strains (Figure S2c) and several of these prophages present a relationship with phages of *Xanthomonas* and *Stenotrophomonas* (Figure S2d). Some inoviruses are present in two copies in the genome as detected in strains Salento-1, Salento-2 and Ann-1, which could suggest superinfection events. *Zonula occludens* toxin (Zot)-like CDSs, a predicted *X. fastidiosa* virulence factor [41,90], were annotated in multiple inoviruses distributed among *X. fastidiosa* strains.

Several groups of genomic islands (GI-G1, GI-G3 and GI-G4) were connected to prophage groups which could be evidence of prophage degradation (Figure 5). A few GIs seem to be related to pathogenicity/virulence or to antibiotic resistance, such as members of GI-G1, GI-G5, GI-G6 and GI-G7, which harbor CDSs encoding hemagglutinin, efflux RND transporter and toxin-antitoxin. Some genomic islands were found in plasmids such as in PLS-G3, PLS-G4, PLS-G5 and PLS-G6 (shown as symbols outlined in dark gray in Figure 5). Like the GI groups mentioned above, the genomic islands of plasmid groups also encode toxin and antitoxin proteins, and in some of these genomic islands, CDSs related to conjugative transfer and type IV system secretion were found (PLS-G4, PLS-G5 and PLS-G6).

Insertion Sequences appear distributed mainly in five clusters with tightly connected nodes (IS-G1 to IS-G5) suggesting that this type of MGE is conserved and could be key in the biology of *X. fastidiosa* species. Other small insertion sequence groups are IS-G6 to IS-G10. Several insertion sequences of the IS-G1, IS-G3, IS-G4 and IS-G5 are found inside other MGEs such as prophages or genomic islands, while other ISs were found in the chromosome. The ISs of IS-G1, IS-G4, IS-G9 and IS-G10 belong to the IS200/IS605 family which is widely spread in Bacteria and Archaea [91]. Members of this family are unusual because they use obligatory single-strand DNA intermediates, which distinguishes them from classical insertion sequences [91]. Other insertion sequence groups were classified into other families such as IS1595, IS21, IS3 and IS6 (IS-G2, IS-G3, IS-G6, IS-G7 and IS-G8 groups), and only two clusters were not classified (IS-G5, and one small cluster at the bottom of the Figure 5).

### 3.6. Immunity Systems Prospection in X. fastidiosa Genomes

**Figure 6:**
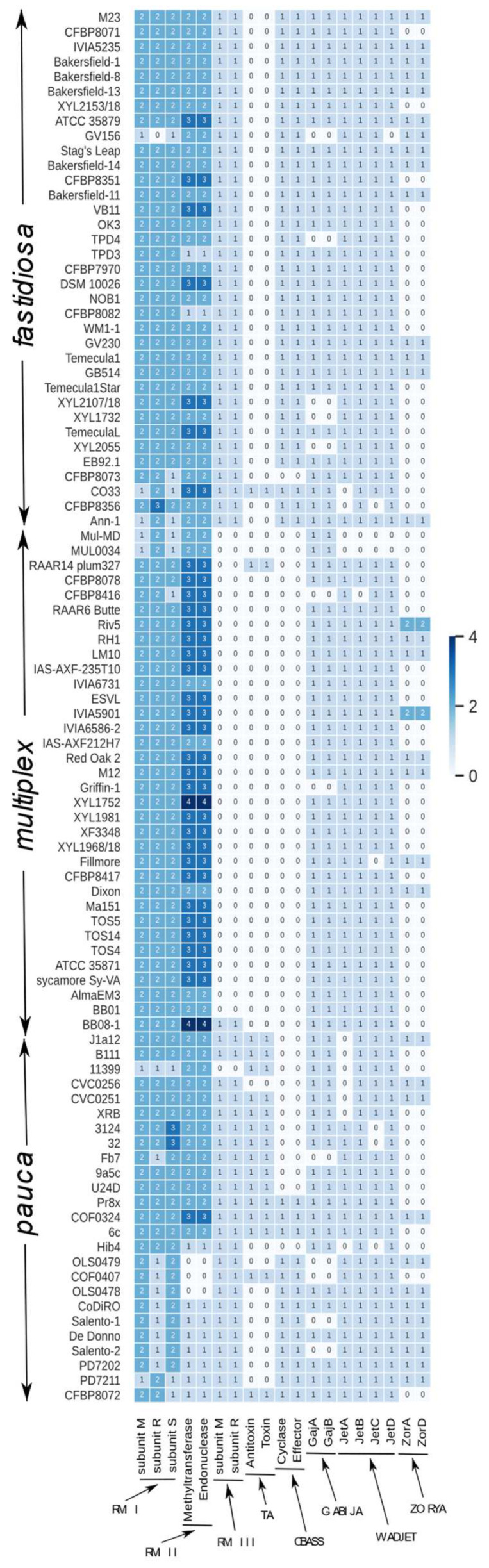
Distribution of the different immunity systems detected in the genomes of *X. fastidiosa* strains.

We performed a screening of the known immunity systems in *X. fastidiosa* to explore the strategies used by this bacterium to deal with their numerous MGEs (Figure 4 and Figure 5). The screening of 94 *X. fastidiosa* genome assemblies (Figure 6) detected only CDSs belonging to Restriction Modification (R-M), Toxin-Antitoxin (TA), Cyclic-oligonucleotide-based antiphage signaling systems (CBASS), Gabija and Wadjet systems. For each detected system, the CDS neighborhood was evaluated. The prediction of R-M systems showed that all strains possess at least one of the three main R-M system types (Figure 6) previously reported for 9a5c and Temecula1 strains [92]. The type II was usually found in multiple operons per genome, while the type III was observed in a single operon per genome. R-M type I and II were frequently found in all strains, and in most instances more than one subunit homolog was observed. In contrast, R-M type III was mainly found among strains of subspecies *pauca* and *fastidiosa*. Curiously, the strains lacking R-M type III (subsp. *multiplex*), have more homologs (4 in some cases) of the R-M type II subunit (Figure 6).

The TA type II system was found mainly in the strains from the subspecies *pauca* from South America. This TA system is widely distributed among prokaryotes and has been confirmed to be involved in diverse biological processes including plasmid maintenance, phage inhibition, stress response, and others [93]. The CBASS phage defense system is composed of an oligonucleotide cyclase, which generates signaling cyclic oligonucleotides in response to phage infection, and an effector that is activated by the cyclic oligonucleotides and promotes cell death [75]. This system was found in strains from the subspecies *pauca* from Europe, and also in strains from the subspecies *fastidiosa*.

The Gabija system is composed of the GajA and GajB proteins that contain ATPase, nuclease and helicase domains [73,94]. Gabija gives immunity to various phages when it was cloned in *B. subtilis*, and to phage T7 when it was cloned in *E. coli* [73]. We found CDS homologs to GajA and GajB in most of the strains except in the complete genomes of Salento-1, Salento-2 and Fb7 strains. It is worth mentioning that in most cases GajA and GajB are separated by a sequence annotated as exodeoxyribonuclease V subunit gamma protein, suggesting this cassette of three CDSs found in *X. fastidiosa* could be a variant of the Gabija system. We detected homologs of ZorA and ZorD that belong to the Zorya antiphage defense system [73]. However, this could not be considered a functional system in *X. fastidiosa* due to the absence of the complete system and its poor conservation among the analyzed genomes. The Wadjet system has been reported to act against foreign plasmidial DNA [73]. Although in some *X. fastidiosa* strains the JatA and JetC are regarded as pseudogenes, in most of the strains the Wadjet genes JetA, JetB, JetC and JetD were found, except in the Mul-MD and MUL0034 strains in which no homolog of these genes is present.

The Abortive infection, pAGOs, DISARM, BREX, HACHIMAN, SHEDU, SEPTU, LAMASSU and DRUANTIA systems [73,95,96,97,98,99] are absent in the 94 *X. fastidiosa* strains analyzed. The same was observed for phage-inducible chromosomal islands (PICI) elements [77]. We also searched for CRISPR-Cas system which could contribute to the prevention of prophage acquisition. Although some Cas homologous CDSs were found, the absence of CRISPR regions suggests that *X. fastidiosa* lacks a functional CRISPR-Cas system as seen for major bacterial lineages [100].

## 4. Discussion

The comparative analyses of 94 publicly available whole-genome sequence assemblies of *X. fastidiosa* strains revealed a pangenome comprising 4549 orthologous CDSs and a core genome of 954 CDSs (Figure 1). These values are somewhat different than previously reported [30,44,45] because we have used different algorithms for genome annotation and clustering of orthologous CDSs as well as a larger number of genomes in the analyses. We found that the vast majority of the CDSs previously identified or predicted to be virulence and pathogenicity factors for *X. fastidiosa* (Table S2) belong either to the core or soft-core genomes.

A core genome-scale phylogeny grouped the 94 *X. fastidiosa* strains in three major clades (Figure 2) defined by strains from the subspecies *fastidiosa* (clade I)*, multiplex* (clade II), and *pauca* (clade III) consistent with previous *k-mers* based phylogeny of 72 *X. fastidiosa* strains [30] as well as with phylogenetic reconstructions from 349 *X. fastidiosa* genomes [86]. While several of the subclades sharing ST groups (mentioned in the Section 3.3) are congruent with country of origin of the strains, plant species from which strains were isolated are less congruent with these subclades. Although some strains isolated from *Citrus*, *Olea*, *Vitis*, and *Morus* group in separated subclades, other strains mainly isolated from *Coffea, Prunus*, and *Nerium* are distributed into the three distinct major clades (Figure 2). It has been shown that citrus and coffee strains from subspecies *pauca* seem to be limited to their original hosts, despite crop proximity and the presence of insect vectors [101,102]. In addition, there is experimental evidence of host specialization for certain *X. fastidiosa* strains [103]. On the other hand, it is known that some strains can infect multiple hosts [10,28,104,105] and that intersubspecific homologous recombination has been associated to *X. fastidiosa* adaptation to novel hosts [25,29,30].

The factors that drive *X. fastidiosa* host-specificity or adaptation to new hosts have not been clearly elucidated [90] despite recent evidence of a genetic basis to the host range of *X. fastidiosa* [86]. Here we have explored the soft-core and core genomes for potential candidates related to this trait using comparative genomics, an approach that has been applied for some bacterial pathogens [106,107,108]. Using a mapping metrics (*Smap*) applied to phylogenetic trees for 1605 orthologous CDSs we found no CDS with *Smap* values that would provide strong support to point a CDS as candidate to host specificity determinant. The highest *Smap* values found were ~0.5, and among these CDSs only a few CDSs were related to virulence, including two related to adhesion (PD0058, PD0528), two related to polysaccharide hydrolysis (PD0529, PD1833), two related to polysaccharide synthesis (PD0815, PD1801) and three encode, respectively, quorum sensing response regulator (PD0406), multidrug efflux pump (PD1403) and lipase/esterase (PD1211) that present medium *Smap* scores. We call attention to CDS PD0815 (Glycosyltransferase family 2 protein) related to LPS biosynthesis. It has been shown that O-antigen delays plant innate immune recognition in grapevine and as such the heterogeneity of O-antigen composition may be related to *X. fastidiosa* host range [36]. In summary, the approach we have used did not provide strong supporting evidence for CDSs that would contribute to *X. fastidiosa* host-specificity. It has been suggested that the *X. fastidiosa* pangenome is linked to host association and the presence/absence of a few genes (mostly encoding hypothetical proteins) in strains isolated specific plant genera have been correlated to host-specificity [86]. However, at the present time some limitations for an experimental study of *X. fastidiosa* host-specificity should be considered such as prompt availability of sequenced isolates as well as the difficult genetic manipulation of some strains [109,110].

Our comparative analyses revealed that the content of MGEs varies among *X. fastidiosa* strains and includes a considerable diversity of sequences related to prophages, GIs, ISs and plasmids with variable sizes (~1.2 kbp to 142.6 kbp). While several MGE sequences are conserved among *X. fastidiosa* strains (e.g., PPH-G8; PPH-G9; IS-G1) some are unique MGEs, belonging to a single strain (e.g Ann-1 prophage and Xfp4 from 9a5c) among the ones we analyzed here. The *X. fastidiosa* 94 genome assemblies harbor 11.6 ± 2.71 prophage-related regions and 6.47 ± 2.57 genomic island regions. A previous study reported 6 and 8 prophage-like elements respectively in genomes of 9a5c and Temecula1 strains [88], and a comparison of 72 *X. fastidiosa* genomes revealed an average of 9.5, 9.3 and 8.5 prophage regions, respectively, for strains from subsp. *fastidiosa*, *multiplex* and *pauca* [30]. It remains to be investigated whether multiple prophage regions confer any fitness advantage to *X. fastidiosa*, as has been observed for *Pseudomonas aeruginosa*, where multiple prophage carriage seems to be beneficial during mixed bacterial infections [111].

It is worth noting that inoviruses sequences [65] are found in most of the analyzed strains (PPH-G8) and that they encode a Zot protein. Inoviruses have a relevant role in the structure in *P. aeruginosa* biofilm [112] and have been reported to encode Zot in several *Vibrio* species [113]. Zot protein seems to play a dual function as it is essential for inovirus morphogenesis and has also been reported to contribute for *Vibrio cholerae* pathogenesis [114,115]. This toxin has been postulated as virulence factor for plant pathogens [116], including *X. fastidiosa* [41,90]. Interestingly EB92-1, a proposed *X. fastidiosa* biocontrol strain, lacks both Zot homologous genes found in Temecula1 strain (PD0915 and PD0928) [117]. Moreover, a *X. fastidiosa* Zot protein was shown to elicit cell death-like responses in the apoplast of some *Nicotiana tabacum* cultivars [34]. Besides Zot, other prophage-encoded genes may play a role in the biology of *X. fastidiosa* as observed in other bacteria, where the so called “moron” loci have been related to virulence, stress resistance, phage resistance and host adaptation [118,119,120]. More studies are necessary to understand the contribution of “moron” loci, such as Zot genes, as well as events of prophage induction to *X. fastidiosa* biology. There is experimental evidence *X. fastidiosa* releases phage particles [88,89,121] but the impact of prophage induction in host colonization is unknown.

To cope with the MGEs, bacteria have developed a diversity of immunity (antiphage defense) systems [73,74,75,95,100]. The numerous immunity systems of some genomes protect the cell from a broad range of MGEs, and the MGEs themselves encode defense systems, which tend to be different across strains of a species [122]. Although *X. fastidiosa* strains are devoid of most of these systems, R-M systems and one conserved cluster with genes of Gabija system [73,94] were found widely distributed among the genome assemblies analyzed in this work. TA type II system [93] and CBASS [75] immunity systems were found only in some strains. It should be mentioned that the R-M systems have been reported to impact the stable acquisition of foreign plasmid DNA by *X. fastidiosa* [92,109]. The low amount and diversity of immunity systems found in *X. fastidiosa* genomes, with the notable absence of important immune systems, especially CRISPR-Cas, gives a hint to understanding the high amount of MGEs found in this bacterial species. It seems that R-M, Gabija and CBASS systems are not enough to protect *X. fastidiosa* against phage acquisition. For instance, Temecula1, one of the most studied *X. fastidiosa* strains, carries 12 prophage regions, but only three immunity systems. This lower amount of immunity systems relative to high number of prophages differs from the positive correlation between the number of prophage and families of antiphage systems observed at species level [123]. Therefore, we do not exclude the possibility that *X. fastidiosa* genomes might encode immunity systems yet to be discovered.

The comprehensive comparative analyses of 94 whole-genome sequences from *X. fastidiosa* strains from diverse hosts and geographic regions contribute to a better understanding of the diversity of phylogenetically close genomes, explores candidates to host specificity determinants for this phytopathogen as well as greatly expands the knowledge of its mobile genetic elements content and of its immunity systems.

## Data Availability

All *X. fastidiosa* genomic sequences were accessed from GenBank RefSeq database at NCBI (National Center for Biotechnology Information). Their respective NCBI accession numbers and publication DOIs are listed in Table S1.

## Supplementary Materials

The following supporting information can be downloaded at: https://www.mdpi.com/article/10.3390/microorganisms10050914/s1, Figure S1: Flowchart describing the workflow implemented to select *X. fastidiosa* GenBank genome assemblies; Figure S2: Selected *X. fastidiosa* prophages groups; Table S1: *X. fastidiosa* genome assemblies publicly available until 2021-07-19 in NCBI Genbank database; Table S2: Predicted virulence and pathogenicity factors for *X. fastidiosa*. Table S3: *Smap* scores for 1605 orthologous CDSs. Table S4: Predicted MGEs in *X. fastidiosa* genome assemblies.
